# The Relationship Between Mindful Agency and Self-Leadership of Chinese Private College Undergraduates: Mediating Effect of Metacognitive Ability

**DOI:** 10.3389/fpsyg.2022.847229

**Published:** 2022-04-12

**Authors:** Zhaojun Chen, Xingxia Zhang

**Affiliations:** ^1^Institute of Education, Xiamen University, Xiamen, China; ^2^College of Humanities, Yantai Nanshan University, Yantai, China; ^3^Graduate School of International Studies, Hanyang University, Seoul, South Korea

**Keywords:** private college undergraduates, self-leadership, mindful agency, metacognitive ability, mediating effect

## Abstract

As one of 21st century key skills, self-leadership is not only the internal factor of private college undergraduates’ independent development, but also related to the quality improvement of talent cultivation of private undergraduate colleges. It is proved that mindfulness or metacognition separately has the predictive effect on self-leadership, but their structural relationships has not been revealed. The present study explored the interrelations between mindful agency, metacognitive ability, and self-leadership through the mediation analysis with structural equation modeling, and bootstrapping was conducted to test the mediating effect. The sample comprised 1,244 private undergraduate sophomore (38.4% male and 61.6% female), and they completed online questionnaires of mindful agency, metacognitive ability, and self-leadership. The results revealed that mindful agency of private undergraduate students not only directly and positively predicted self-leadership, but also indirectly and positively predicted self-leadership through the mediating effect of metacognitive ability. Metacognitive ability partially mediated the relationship between mindful agency and self-leadership. The direct effect of mindful agency and the mediating effect of metacognitive ability, respectively, account for 86.9% and 13.1% of the total effect. The results suggest that the more mindful private college undergraduates are, the more willing they are to practise their metacognitive skills in their learning, and the more progress in self-leadership they make. Educational implications for mindfulness training and metacognition practice to foster their self-leadership are discussed.

## Introduction

In 2019, the gross enrollment rate of Chinese higher education reached 51.6%, indicating that Chinese higher education has been in the stage of popularization, according to the statistical bulletin on the development of national education released by Chinese Ministry of Education (2020). Private undergraduate education as “half the sky” of overall undergraduate education in China, not only plays an indispensable role in the transition from mass higher education to popular higher education, but also is related to the high-quality development of overall Chinese undergraduate education (Luo et al., 2020). Chinese private undergraduate colleges are run by social forces, started late with a weak running foundation, and satisfied the desire of many young people to go to college and the social demand for application-oriented talents, but the quality construction of talent cultivation is still on the way (Pan et al., 2018). Since 1994, when China’s first private undergraduate university (Yang-En University) was founded, for the purpose of survival, private undergraduate colleges attach more importance to the scale expansion and enrollment work than talent cultivation quality management so that their education quality became inferior in the 20th century. Even if their undergraduate education quality were improved in the early 21st century, but still in the “lowland” (Que et al., 2019). Compared with public undergraduates, private undergraduates with lower college entrance examination scores generally show lower self-regulation and learning confidence, and even escape learning (Chen and Xiong, 2013; Shen and Wang, 2015). If private undergraduates’ these problems are not solved in time, their talent quality will be directly affected.

At present, the 21st century skills-oriented educational reform movements beyond knowledge are undergone in countries around the world, emphasizing the coordinated development of individual cognitive, intrapersonal and interpersonal skills (National Research Council, 2012). Locus of control theory holds that individuals with a high internal locus of control not only believe in their own ability to control themselves and influence others, but also are more confident, motivated and success-oriented (Rotter, 1990). As an important “internal locus of control” of individual development, self-leadership is one of the 21st century key skills, and can promote the enhancement of individual self-control and self-confidence (Manz, 2015) and the realization of target performance (Neck et al., 2003). Moreover, it is beneficial to the progress of individuals and groups (Neck and Houghton, 2006) and the improvement of college student’ civic consciousness, social responsibility and innovation ability (Komives and Wagner, 2009; Zhao and Zhou, 2017).

Self-leadership is not inborn but nurtured. Its development is based on individual’s self-cognition and self-regulation (Godwin et al., 1999). Mindfulness means a individual’s self-awareness of the present moment purposefully and non-judgmentally. As a way to self-regulation, mindfulness is rooted in the practice in Eastern Buddhism. In 1979, Dr. Jon Kabat-Zinn introduced mindfulness from Buddhism to clinical applications and developed Mindfulness-Based Stress Reduction (MBSR) program to treat chronic pains and deeper distress caused by patients’ attempt to avoid pain (Baer, 2003; Kabat-Zinn, 2003), which contributed to the emergence and development of mindfulness in Western culture. Just like MBSR, mindfulness-based cognitive therapy was also confirmed to be effective for reducing anxiety and distress (Hofmann et al., 2010). Mindfulness plays both direct and indirect roles in lowering adults’ level of depression and anxiety (Parmentier et al., 2019). As a positive psychology factor, mindfulness is beneficial for self-determination and is often taken as the key path to improving individuals’ subjective happiness and self-actualization (Seligman, 2002; Brown and Ryan, 2003; Beitel et al., 2014; Whitehead et al., 2020). For purpose of the mental health and development of students, mindfulness meditation, characterized by being evidence-based, self-guided and easily accessible, and even low-cost or free, is also introduced into educational fields. For example, Su and Shum (2019) demonstrated that mindfulness might prevent adolescent students’ possible psychological distress related to critical thinking which is widely considered as an essential cognitive skill in the twenty-first century. Mindfulness in the classroom is qualified for promoting students’ learning concentration and social emotional learning (Armstrong, 2022) that is carried out to develop learners’ self-awareness, self-management, social awareness, relationship skills and responsible decision-making (Osher et al., 2016). Students’ effectively regulating their emotions and making responsible decision about their prosocial behavior. Students’ effectively regulating their emotions and showing their prosocial behavior lies in the relevance of mindfulness to metacognition. There are discussed to be different levels of metacognition in mindfulness during which individuals consciously establish metacognitive experiences and unconsciously monitor processes (Jankowski and Holas, 2014). Mindfulness is also conceptualized as metacognitive practice (Kudesia, 2019), that is, adjusting individuals’ mode of information processing to their present moment and helping them achieve greater agency and resilience in their responding to the current situation. Metacognitive skills, such as self-awareness, regulation and monitoring, can support giftedness in leadership (Kontostavlou and Drigas, 2021). Mediating effect of metacognition has recently been proven in the relationship between mindfulness and some variables such as critical thinking (Soliemanifar et al., 2021), but no relevant empirical research demonstrates mediating effect of metacognition in the relationship between mindfulness and self-leadership.

It is proved that mindfulness (Sampl et al., 2017; Furtner et al., 2018) or metacognition (Black et al., 2016; Kontostavlou and Drigas, 2021) is beneficial for the development of individuals’ self-leadership. Actually speaking, there is no relevant empirical research on the internal structural relationship among these three variables (mindfulness, metacognition and leadership) in education or university related fields. In China, the necessity to improve the self-leadership of students in private colleges and universities is highly emphasized so as to meet the needs of self-actualization and social development in the new era, the internal structural relationship among mindfulness, metacognition and leadership needs to be revealed so that effective educational measures could be guided and taken. Accordingly, it is of great significance to study the self-leadership and influence mechanism of private undergraduates in order to promote their academic development and improve the quality of talent cultivation in private undergraduate colleges or universities.

## Literature Review and Research Hypothesis

### Self-Leadership

The concept of self-leadership first appeared in the field of management, first proposed by Manz and Sims (1980). Subsequently, Manz (1986) defined self-leadership as the process affecting individuals to establish and execute their self-direction and self-motivation. Later, Neck and Manz (2004) gave an authoritative and widely recognized definition, that is, self-leadership is a behavioral process of self-assessment and self-influence, and a positive behavioral process in which people improve their overall performance through self-direction and self-motivation. To measure individuals’ leadership, Anderson and Prussia (1997) developed 50-item Self-Leadership Questionnaire (SLQ) based on self-leadership’s three frameworks of behavior-focused strategies, natural reward strategies and constructive thought pattern strategies. Houghton and Neck (2002) revised the 50 items of SLQ into 35 items and made the Revised Self-Leadership Questionnaire (RSLQ), including 9 dimensions: (1) self-observation (focusing on the occurrence of one’s own behavior), (2) self-goal setting (effective guidance for self-behavior management), (3) self-reward (combined with self-goal setting to motivate individuals to work hard to achieve set goals), (4) self-punishment (introspection and feedback to be closer to the positive expected results), (5) self-cueing (informing to engage in positive behavioral activities and avoid negative ones), (6) natural reward (finding pleasure in a given task or behavior so as to improve self-control and achieve goals), (7) self-talk (positive psychological hints for eliminating negative emotion and anxiety), (8) evaluating beliefs (accuracy and positive influence of current beliefs), and (9) visualizing success (mental picture of oneself as a success). RSLQ was widely used in the field of management research focusing on the impact of self-leadership on individuals and teams, and the role of self-leadership in promoting the development of individuals and teams. For example, it was found that employees’ self-leadership positively affected the development of their creativity (Carmeli et al., 2006; Amundsen and Martinsen, 2015), and their task performance and proactive behaviors (Qian et al., 2018), and on the basis of cultural psychology, leaders’ better leadership can reduce employees’ counterproductive work behavior (Shen and Lei, 2022).

Since the 1980s, American universities started leadership education for college students, such as Yale University and Oxford University. More than 1,000 universities are carrying out leadership education (Komives and Wagner, 2009), taking civic awareness, social responsibility, innovative spirit and problem-solving capability as the cores of leadership education of college students. In 1996, the Higher Education Research Institute of University of California (Los Angeles) proposed the most influential model of college students’ Socially Responsible Leadership whose core content emphasizes individuals’ abilities of self-cognition, working cooperatively, focusing on community responsibility and public common interests (Higher Education Research Institute, 1996). In addition, some studies revealed there was the gender effect on self-leadership. A meta analysis demonstrated that men tended to rate their better leadership than women on the account of expected gender roles, but other-ratings were superior for women’s leadership to men (Paustian-Underdahl et al., 2014). College students’ leadership development also benefits from their personality traits. Huszczo and Endres (2017) took 325 college business students as samples, and found that women with higher conscientiousness level than men, had more leadership self-efficacy, and by contrast, extraversion was more predictive of leadership efficacy for men than women. As for Chinese undergraduate student, empirical researches show that in general, girls’ self-leadership level is significantly higher than boys, because compared with boy, girls are generally more careful, better in self-control, more aware of competition, and have a stronger sense of social responsibility to themselves, others, and collective (Chen et al., 2014; Liang et al., 2015).

College students’ self-leadership can be regarded as a tripartite construction of relationships with self, with others and with the collective. Tripartite model of self-construal holds that individual self (for uniqueness and independence), relational self (for interpersonal attachments with close others and interdependence), and collective self (for membership in and belonging to core social groups) coexist in one individual and these three kinds of self-construal are the basic psychological needs of human beings in social relations (Brewer and Gardner, 1996; Sedikides and Brewer, 2001). Brown and Ryan (2003) believed that one of the preconditions to meet the basic psychological needs of the self is that individuals must have a keen perception of their own needs and the current environment, so as to more accurately choose behaviors that can meet the needs of autonomy (independence), competence (interdependence) and relatedness (belonging), and engage in the activities with a positive perception. The perception and regulation of independence is the most basic value in leadership development, and it is accurate reflection of current behaviors and mentality of self, representing the mindfulness (Tao, 2015; Wang and Peng, 2017).

### Mindfulness and Self-Leadership

Mindfulness is an individual’s experience of purposefully focusing on the present moment with an open, accepting, and balanced attitude (Kabat-Zinn, 1982). It is a state of consciousness that occurs by directing attention to the present experience without judgment (Kabat-Zinn, 2003). Mindfulness enables individuals to focus purposefully on the present situation, without making any judgments or reactions, and fully focus on their own experience. In the field of management, studies have shown that mindfulness as a key factor of psychological regulation positively predicts managers’ self-leadership (Furtner et al., 2018). Mindfulness is the psychological premise of individual leadership development. It is proven that mindfulness can lead to high-quality relationships, emotional mastery, and more openness to new ideas (Dutton and Heaphy, 2003; Fletcher, 2007), and has a significant impact on the improvement of leadership effectiveness (Wasylkiw et al., 2015). In the field of educational psychology, learners can improve their ability to regulate their emotions and monitor their learning cognitive process through the method of mindfulness, so as to form mindful agency that means learners can adjust their emotions and plan their learning process independently with a high sense of responsibility for learning (Wang and Peng, 2017). Based on previous studies, Wang and Peng (2017) further developed and verified Mindful Agency Inventory for Chinese College Students, including five factors: (1) learning methods investigating students’ awareness of finding and mastering methods in problem solving; (2) emotional regulation investigating if students are self-motivated and adjusted when their learning emotions are not strong or motivation is weak; (3) awareness of planning investigating students’ choice of learning strategies and planning awareness of learning; (4) openness to experience investigating students’ openness to learning experiences and intuitions in the learning process; and (5) learning engagement investigating if students are absorbed in the learning process and focus their attention on the present moment. This framework of Chinese college students’ mindful agency is advised to be applied to the study on Chinese college student development.

Some studies revealed the relationship between mindfulness and self-leadership. In the management science, mindfulness and leadership have been integrated into mindful leadership for leaders (Peng et al., 2019; Rupprecht et al., 2019; Shen and Ge, 2021). In the field of modern educational psychology, a narrative-oriented inquiry of undergraduates suggests that mindful agency is a positive learning disposition, and enhances learners’ self-awareness and self-awakening (Wang et al., 2017). A psychological intervention experiment of college students shows that the success rate of mindfulness training in improving college students’ self-awareness, self-management and interpersonal skills accounts for 78% (Conley, 2015), and mindfulness can effectively strengthen undergraduate students’ self-leadership, and the effect of mindfulness on self-leadership is long-term (Sampl et al., 2017). Therefore, mindfulness agency can satisfy the demand premise of the college students’ leadership development, and help college students actively and consciously perceive and monitor their self-leadership development.

*Hypothesis 1: Mindfulness agency of private undergraduate students can directly predict their self-leadership.*

### Metacognitive Ability and Self-Leadership

Metacognition refers to the individual’s knowledge and awareness of its own cognitive process, and on this basis, the individual’s self-reflection, self-evaluation and self-regulation of the cognitive process (Flavell, 1992). In essence, metacognition refers to people’s self-awareness, self-control and self-regulation of cognitive activities, including metacognitive knowledge, metacognitive experience and metacognitive monitoring, which restricts the development level of individual intelligence and thinking (Wang, 2004). Based on previous studies, Kang (2005) developed and verified metacognitive ability inventory for Chinese college students, which included four factors: (1) metacognitive planning investigating students’ perception and understanding of their own cognitive ability, cognitive strategies and cognitive tasks; (2) metacognitive monitoring investigating students’ identifying tasks, evaluating and predicting cognitive progress; (3) metacognitive regulating investigating students’ configuring learning resources and deploying the steps and pace of task completion; and (4) metacognitive evaluating investigating students’ finishing the cognitive task, and judging their own cognitive effect and stimulating the motivation to continue learning.

Metacognition is an individual’s ability to monitor and control cognitive states (Goupil and Kouider, 2019; Heyes et al., 2020). Metacognition plays an active role in individual cognitive activities and has five educational functions in individual development, namely, promoting individual intelligence development, improving the efficiency of achieving cognitive goals, enhancing learning ability, making up for general cognitive ability, and cultivating students’ subjectivity (Wang, 2004). Furthermore, metacognition can correct cognitive bias and realize cognitive reconstruction, help individuals to get away from problems and review and solve problems from a bystander’s perspective (Connell and Wellborn, 1991), which is conducive to the development of individual autonomy. Black et al. (2016) believe that metacognition promotes the development of leadership by monitoring and adjusting incongruous concepts in individual cognition and improving self-awareness, self-learning management, understanding and influencing others. Therefore, metacognition can help college students build the ability of self-education, self-management and self-perfection. Metacognitive ability is helpful for college students to change their thinking patterns and revise their self-perception of leadership.

*Hypothesis 2: Metacognitive abilities of private undergraduate students affect their self-leadership development.*

### Mindfulness Agency and Metacognitive Ability

As a positive learning psychological quality, mindful agency can reduce the level of negative emotional factors such as anxiety and depression, and improve the level of positive emotional factors such as happiness, self-confidence, optimism, and psychological resilience (Brown and Ryan, 2003; Liu et al., 2015). The fundamental reason why mindfulness promotes positive psychology lies in its regulation of metacognition. Studies have shown that mindfulness can enable learners to regain awareness of their internal behaviors through deautomatization, thus reducing the habituation of cognitive behaviors (Deikman, 2000; Wang et al., 2012). Mindfulness agency is helpful for learners to increase their emotional intelligence, self-efficacy and even metacognition and to be self-directed and self-determined (Peng and Wang, 2019; Wang and Lu, 2020). By practicing mindfulness meditation, college students changed their cognitive bias, reduced anxiety and negative emotions, improved their sense of hope (Sears and Kraus, 2009), and hence adapted to stressful events in life and increased happiness, and also further promoted the improvement of metacognitive abilities (Garland et al., 2009). Therefore, mindfulness not only directly regulates self-leadership development but also affects metacognitive ability.

*Hypothesis 3: Mindful agency of private undergraduate students can indirectly predict their self-leadership, and metacognitive ability plays a mediating role in the relationship between mindful agency and self-leadership.*

**FIGURE 1:**
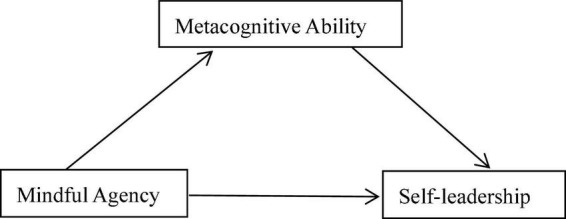
Hypothesis model.

To sum up, it is of great significance to study the self-leadership and its internal influencing mechanism. However, there is no empirical study on the relationship among mindfulness agency, metacognitive ability and self-leadership among private undergraduate students. This study attempts to explore the influence mechanism of mindfulness agency and metacognitive ability on self-leadership of private undergraduate students through questionnaire survey. Based on the 3P model of “presage-process-product” proposed by Biggs (1993), this study constructed a hypothesis model with the mindfulness agency as the presage variable, metacognitive ability as the process variable, and self-leadership as the product variable, as shown in Figure 1.

## Materials and Methods

### Participants

In this study, sophomores are chosen as participants, who basically adapt to college life after freshman year, but they are prone to slacking in learning. Generally speaking, the second year of college students is the “low tide” of learning (Lv, 2015). Studies have shown that students who have completed the transition are capable of significantly improving their academic performance and comprehensive competence, while those who have not completed the transition are at a disadvantage in the academic achievement and competence development (Yang et al., 2013). In view of this, it is necessary to study and improve private undergraduate students’ self-leadership to enhance their learning motivation and to lay a solid psychological foundation for coping with the coming academic pressure.

Participants were 1,244 sophomores from two full-time private undergraduate colleges in mainland China, with a mean age of 18.9 years (±1.3). All subjects in this study have not any neuro developmental disorder such as attention deficit hyperactivity disorder (ADHD). An online questionnaire was formed through the network platform. Each item was set as a required answer, and students were voluntary to answer all the questions before submitting the questionnaire to ensure that there was no omission in the answers. They were invited to log in the online survey platform and answer the online questionnaire anonymously, and they typically spent 8 min. The saved data were all kept confidential. Of the sample, males accounted for 38.4% (478) and females for 61.6% (766). As to discipline, 50.5% (628) of participants major in liberal arts, and 49.5% (616) in science and engineering.

## Data Analysis

In the data measured by the self-report scale, data collection methods, characteristics of the item itself, the response bias of participants, etc., may cause common method bias (CMB) influencing the measurement validity (Podsakoff et al., 2003). Before the formal analysis of the data, this study adopted Harman’s single-factor test method (Zhou and Long, 2004) to assess the common method bias since the data for all the measures was collected with a single questionnaire method in this study. The single-factor model fit indices were as follows: x^2^/df = 41.96, CFI = 0.78, NNFI = 0.73, GFI = 0.70, RMSEA = 0.18, indicating that the model fit was not good, and there should not be a serious common method variance in this study.

IBM SPSS25.0 was used to conduct descriptive analysis and correlation analysis. IBM AMOS21.0 was used for confirmatory factor analysis (CFA) to assess the reliability and validity of the measurement models, and more importantly to confirm the theoretical construction.

Mediation analysis is necessarily performed to explain the mechanism through which mindful agency (independent variable) influences self-leadership (outcome) under the mediating effect of metacognitive ability (mediator), so as to enlighten us to explore future efficient intervention measures. Structural equation model for the theoretical construction, represented by path diagrams, can be used to perform mediation analysis, and also to provide mediation model fit indexes for the consistency of hypothesis to the data in this study.

In the mediating effect test, bootstrap method is a resampling method which generates bootstrap replicate samples with replacement from our original sample by randomly choosing among the original sample. It is used to compute a usually 95% confidence interval (CI) to verify the significance of the mediating effect. If the 95% CI does not contain 0 between the lower limit and the upper limit, the effect is significant.

## Measures

### Mindful Agency

A 16-item mindful agency inventory developed and verified by Wang and Peng (2017), was used to measure college students’ evaluations of their mindfully learning abilities. Five scales were extracted from this inventory, including learning methods (an example item “I knew that if something was important to learn, I would find a way to learn it”), emotional regulation (“When I’m frustrated with my learning, I’m good at finding ways to feel like learning”), awareness of planning (“I can usually predict how long it will take me to learn something”), openness to experience (“Some good ideas came into my mind in a casual way”), and learning engagement (“I can get so absorbed in what I’m doing that I forget the passage of time”). Items are rated on a five-point Likert score ranging from “1 = strongly disagree” to “5 = strongly agree.” As for the reliability analysis, Cronbach’s α value for this inventory in this study is 0.91. Besides, as proposed by Fornell and Larcker (1981), the composite reliability (CR) of one construct is determined to ensure the internal consistency. The composite reliability of the inventory in this study is 0.81. Thus, the internal consistency of this inventory is confirmed to be high. The confirmatory factor analysis results were as the following: x^2^/df = 6.98, CFI = 0.99, NNFI = 0.97, RMSEA = 0.069. In the structure equation modeling with AMOS, Byne (2001) points out that if NNFI and CFI are greater than 0.9 and RMSEA are less than 0.08, data fit is acceptable. In this study, this inventory can better evaluate Chinese private undergraduate students’ mindful agency with good reliability and validity.

### Metacognitive Ability

A 24-item metacognitive ability inventory developed and verified by Kang (2005) was used to measure college students’ evaluations of their awareness of cognitive learning abilities. Four scales were extracted from this inventory, including metacognitive planning (“Before I work on a task, I set some clear goals”), metacognitive monitoring (“I can feel that I’m thinking.”), metacognitive regulating (“When I do something, I think about what I really need to learn”), and metacognitive evaluating (“After I do something, I think about whether I have really learned what I needed to learn”). Items are rated on a five-point Likert score ranging from “1 = never” to “5 = always.” Cronbach’s α of the inventory in this study is 0.95, and CR is 0.92, both of which show the internal consistency is high. The confirmatory factor analysis results were as the following: x^2^/df = 2.7, CFI = 0.99, NNFI = 0.99, RMSEA = 0.04. In this study, this inventory can better evaluate Chinese private undergraduate students’ metacognitive ability with good reliability and validity.

### Self-Leadership

The 35-item self-leadership inventory is the Chinese version of Houghton and Neck’s Revised Self-leadership Questionnaire (RSLQ) translated by Wang (2014). Nine scales were extracted from this inventory, including self-observation (“I keep track of the pace of my current tasks”), self-goal setting (“I set specific goals for my tasks”), self-reward (“When I successfully complete a task, I often reward myself with something I like”), self-punishment (“I feel guilty when I don’t perform well on a task”), self-cueing (“I keep a journal to remind myself of the tasks I need to complete”), natural reward (“In the task, I tend to look for activities that interest me “), visualizing success (“I imagine myself performing well on important tasks”), evaluating beliefs (“Whenever I face difficult times, I will keep faith and hope”), and self-talk (“When faced with difficulties, I will communicate with myself how to overcome them”). Items are rated on a five-point Likert score ranging from “1 = strongly inconsistent” to “5 = strongly consistent.” In this study, Cronbach’s α of this inventory is 0.94, and CR is 0.91, indicating the internal consistency is high. The confirmatory factor analysis results were as the following: x^2^/df = 7.39, CFI = 0.97, NNFI = 0.96, RMSEA = 0.072. In this study, this inventory can better evaluate Chinese private undergraduate students’ self-leadership potentials with good reliability and validity.

## Results

### Description and Correlation

Table 1 summarizes descriptive statistics and correlation matrices for each variable. In terms of mindfulness agency, students’ scores in the five are all higher than the theoretical median of three points, indicating that private undergraduate students have positive learning psychology in self-regulation with mindfulness. They perform better in learning methods (*M* = 3.71), but there is still room for further improvement in their emotional regulation (*M* = 3.37) and awareness of planning (*M* = 3.50). As for metacognitive ability, they have the better performance in metacognitive evaluating (*M* = 3.63), but lower in metacognitive regulating (*M* = 3.53) and planning (*M* = 3.50). According to reported 9 indicators of self-leadership, students have good potentials for self leadership development in which they report the better scores in self-goal setting (*M* = 3.79), evaluating beliefs (*M* = 3.78), self-reward (*M* = 3.77), and natural reward (*M* = 3.76), but their self-cueing (*M* = 3.33) needs improving. In terms of correlations between variables, there were significant positive correlations among all variables (*ps* < 0.01). Most variables of mindful agency and metacognitive ability present (1) minimal positive correlations (0 < *rs* < 0.3, *ps* < 0.01) with self-reward and self-punishment, (2) weak positive correlation (0.3 < rs < 0.5, *ps* < 0.01) with self-cueing, self-talk, self-reward, and visualizing success, and (3) medium positive correlation (0.5 < rs < 0.7, *ps* < 0.01) with self-observation, self-goal setting, and evaluating beliefs in self-leadership variable.

**TABLE 1:** Correlation matrix and descriptive statistics of mindful agency, metacognitive ability and self-leadership (*n* = 1,244).

Factors	M	SD	1	2	3	4	5	6	7	8	9	10	11	12	13	14	15	16	17	18
1. learning methods	3.71	0.60																		
2. emotional regulation	3.37	0.72	0.47**																	
3. awareness of planning	3.50	0.60	0.48**	0.55**																
4. openness to experience	3.55	0.55	0.47**	0.44**	0.60**															
5. learning engagement	3.68	0.63	0.41**	0.28**	0.39**	0.55**														
6. metacognitive planning	3.54	0.62	0.45**	0.41**	0.55**	0.51**	0.31**													
7. metacognitive monitoring	3.57	0.58	0.48**	0.36**	0.51**	0.52**	0.36**	0.73**												
8. metacognitive regulating	3.53	0.66	0.44**	0.44**	0.53**	0.50**	0.31**	0.73**	0.76**											
9. metacognitive evaluating	3.63	0.62	0.46**	0.36**	0.49**	0.50**	0.37**	0.70**	0.74**	0.81**										
10. self-observation	3.68	0.55	0.53**	0.42**	0.50**	0.51**	0.42**	0.50**	0.48**	0.51**	0.50**									
11. self-goal setting	3.79	0.60	0.59**	0.49**	0.53**	0.50**	0.39**	0.60**	0.54**	0.57**	0.56**	0.74**								
12. self-reward	3.77	0.78	0.29**	0.20**	0.22**	0.25**	0.26**	0.22**	0.24**	0.23**	0.25**	0.40**	0.38**							
13. self-punishment	3.53	0.57	0.32**	0.21**	0.23**	0.32**	0.33**	0.17**	0.21**	0.21**	0.23**	0.47**	0.36**	0.37**						
14. self-cueing	3.33	0.80	0.31**	0.36**	0.36**	0.31**	0.22**	0.40**	0.32**	0.38**	0.34**	0.50**	0.54**	0.31**	0.25**					
15. natural reward	3.76	0.56	0.58**	0.40**	0.44**	0.49**	0.45**	0.44**	0.46**	0.44**	0.48**	0.68**	0.66**	0.46**	0.41**	0.42**				
16. visualizing success	3.61	0.57	0.51**	0.38**	0.47**	0.48**	0.43**	0.46**	0.48**	0.45**	0.44**	0.65**	0.65**	0.46**	0.46**	0.40**	0.66**			
17. evaluating beliefs	3.78	0.57	0.56**	0.45**	0.51**	0.53**	0.44**	0.52**	0.54**	0.51**	0.53**	0.72**	0.74**	0.48**	0.44**	0.43**	0.68**	0.65**		
18. self-talk	3.70	0.68	0.44**	0.38**	0.38**	0.41**	0.38**	0.42**	0.43**	0.43**	0.44**	0.61**	0.63**	0.43**	0.38**	0.38**	0.56**	0.58**	0.64**	

**p < 0.01, M for Mean, SD for Standard deviation.

### Mediating Effect Test

**FIGURE 2:**
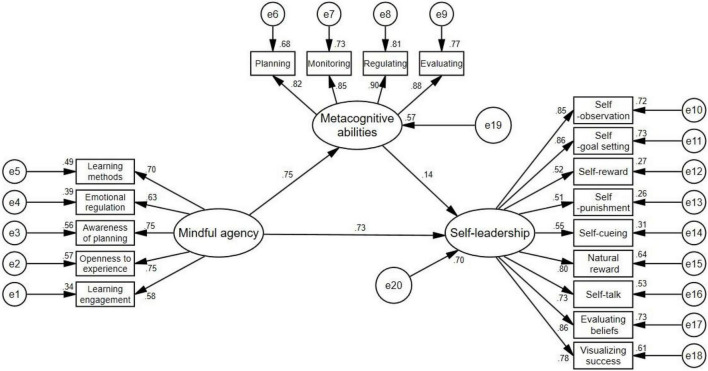
Structural equation model of mindful agency, metacognitive ability and self-leadership (*n* = 1,244).

Structural equation model in Figure 2 shows the analysis of relationships among mindful agency, metacognitive ability, and self-leadership. This model had acceptable fit indexes (x^2^/df = 7.37, CFI = 0.94, NNFI = 0.93, RMSEA = 0.072). As shown in Figure 2, mindful agency directly positively predicts self-leadership and indirectly positively predicts self-leadership through the mediation role of metacognitive ability. To be more specific, regarding the direct relationships, mindful agency positively predicts metacognitive ability (β = 0.75, *p* = 0.001 < 0.01) and self-leadership (β = 0.73, *p* = 0.001 < 0.01). Metacognitive ability positively predicts self-leadership (β = 0.14, *p* = 0.019 < 0.05). With respect to the indirect effect, metacognitive ability mediated the relationship between mindful agency and self-leadership (β = 0.11, CI: 0.03−0.18, *p* = 0.001 < 0.01).

Bootstrapping procedures (resampling the data multiple 2,000 times) were adopted to test the mediating effect of metacognitive ability. The 95% bootstrapped confidence interval (CI) of the mediating effect did not include zero, showing the significance of metacognitive ability’s indirect effect. The direct effect and total effect of mindfulness agency on self-leadership were 0.73 and 0.84, respectively. The partial mediating effect of metacognitive ability accounted for 13.1% of the total effect, and the direct effect of mindfulness agency accounted for 86.9% of the total effect. The variance accounted for by the predictor variable in this model was *R*^2^ = 0.57 for metacognitive ability (95% CI: 0.51−0.63, *p* = 0.001 < 0.01), *R*^2^ = 0.70 for self-leadership (95% CI: 0.65−0.76, *p* = 0.001 < 0.01).

## Discussion

Based on the analysis of 1,244 student samples from private undergraduate colleges, this study constructs a structural equation model to study the relationship among mindfulness agency, metacognitive ability and self-leadership. As expected, the results in the present study indicated private undergraduate students’ mindful agency, metacognitive ability and self-leadership were positively correlated with each other, and their structural relationships were revealed by means of the SEM analyses. Metacognitive ability can partially mediate the relationship between mindful agency and self-leadership. The mediating effect of metacognitive ability was confirmed by the path model. Mindful agency can directly predict self-leadership and also indirectly predict self-leadership by means of metacognitive ability.

### Students’ Development of Self-Leadership

The descriptive results of this study show that among factors of self-leadership, students are better at self-goal setting (*M* = 3.79), that is, they have effective guidance for self-behavior management, which is consistent with the research result of Wang (2014) on college students in ethnic areas. To a certain extent, behaving rules required in Chinese traditional family and school education encourage students to set goals. Data also shows that students pay more attention to self-reward (*M* = 3.77), natural reward (*M* = 3.76) and evaluating beliefs (*M* = 3.78), all of which are related to students’ successfully completing a task. They would reward themselves with things they liked, evaluate their own beliefs and assumptions, and reflect on difficulties they have met.

Private undergraduate students perform not well in self-cueing (*M* = 3.33), which is consistent with the research results of Wang (2014). Private undergraduate students perform insufficiently in using something to remind themselves of tasks they needed to complete, which indicates they tend to repeat behaviors they are familiar with and good at, and less actively engage in creative thinking. Their overall self-leadership has not reached a high level, for two main reasons. On one hand, their self-discipline and self-control are not strong (Chen and Xiong, 2013; Shen and Wang, 2015). On the other hand, their mindful agency and metacognitive ability are not at a higher level. Studies have shown that mindfulness is beneficial to improve self-leadership efficacy (Wasylkiw et al., 2015), and metacognitive ability helps to change individuals’ stubborn thinking and correct their cognitive distortion (Connell and Wellborn, 1991). If private undergraduate students improve their development of mindful agency and metacognition, they will deepen their understanding of self-leadership knowledge and strategy application. For example, they are aware that they need to strengthen their self-cueing behavior by means of journals, so as to change the habitual and rigid thinking modes.

### Effects on Students’ Development of Self-Leadership

With respect to the direct effect in this study, mindfulness agency of private undergraduate students directly positively predicts their self-leadership. The stronger the students’ mindful agency is, the higher level their self-leadership will get to, which is in line with previous researches (Brown and Ryan, 2003; Wasylkiw et al., 2015; Sampl et al., 2017). Mindful agency can satisfy the psychological premise needs of individuals’ self- leadership and is a key predictor of their self-leadership development.

As to the indirect effect, students’ metacognitive ability displays the mediating effect between mindful agency and self-leadership. The more mindful agency students have, the more perception of learning environments students are engaged in, and students are more willing to apply their metacognitive ability to their learning process, make self-assessment and self-adjusting for their cognitive objectives, and meantime promote their awareness of self-leadership. Thus, their self-leadership will be further enhanced. Garland et al. (2009) pointed out the role of mindfulness in positive reappraisal. Mindfulness with the help of metacognition turns the initial stress assessment into a positive reassessment that is necessary to the establishment of self-orientation and self-motivation in the process of self-leadership development (Manz, 1986). Mindfulness emphasizes being aware of one’s feelings and thoughts in the present moment. This positive perception of the present stimulates the essential functions of metacognition, that is, individuals’ self-awareness, self-reflection and self-regulation. Metacognition promotes the development of individuals’ self-leadership by enabling individuals to recognize their own advantages and disadvantages, understand the known and unknown, and monitor and adjust their learning needs (Avolio and Hannah, 2008).

In general, compared with previous studies, this study is characterized by a more comprehensive focus on the relationship between mindful agency, metacognitive ability and self-leadership. Most previous studies only explored the relationship between the two variables, such as mindfulness and self-leadership (Sampl et al., 2017; Furtner et al., 2018), mindfulness and metacognitive ability (Garland et al., 2009; Sears and Kraus, 2009), metacognition and self-leadership (Black et al., 2016). Therefore, this study can comprehensively reveal the internal relationship among mindful agency, metacognitive ability and self-leadership.

### Theoretical Implications on Learners’ Mindful Self-Leadership

In this study, the confirmed construction relationships indicate some theoretical implications on mindful self-leadership in the field of learning psychology. At present, mindful leadership is used in workplaces to improve leaders’ management and leadership by enhancing their self-perception, self-regulation, and self-reflection through mindfulness training (Peng et al., 2019; Shen and Ge, 2021). Leaders need mindful leadership in work, and it is also true of learners in college. In learning activities, if learners have the stronger mindful agency, their metacognitive ability will be better improved, and next their self-leadership can be further enhanced. Individuals’ metacognitive ability not only contributes to the impact of their mindful agency on their self-leadership, but also deepens the connectedness between mindful agency and self-leadership. Individuals with strong mindful agency often focus on the present learning and perceive their internal learning experience and their emotion as an observer, use their metacognition to strengthen their self-awareness and self-management of the current learning environment, and comprehensively think over and regulate present problems, so as to impose their mindfulness on their self-leadership. Once mindful agency and self-leadership are integrated through the medium of metacognition, mindful self-leadership will be achieved. Eventually, mindful self-leadership come into being in individuals’ learning. As Rupprecht et al. (2019) found, mindfulness training caused changes in leaders’ cognitive nervous system, their behaviors were further affected, and their self-leadership was improved. On that account, leaders’ inclusive leadership (Fang et al., 2019, 2021) was also promoted, and their employees’ failures would be understood and tolerated. In the same way, mindfulness self-leadership enables learners to focus on and be sensitive about the learning environment and make inclusive decisions considerately and comprehensively about their learning engagement and even learning failure.

### Suggestions on Improving Students’ Self-Leadership

According to the results of this study, the stronger mindful agency of private undergraduate students is, the higher level their metacognitive ability is at, and the better their development of self-leadership is. Private undergraduate education workers should give full play to the role of mindfulness intervention, develop personalized mindfulness training programs, relieve private undergraduate students’ academic and psychological pressure, improve their awareness of self-discipline and self-management. As the meta-analysis review provides, effectiveness of mindfulness in reducing anxiety and depression, increasing well-being, and improving performance is evident (Hofmann et al., 2010; Hoffman and Gomez, 2017). For the individual development, as it is indicated in an empirical research, college students’ mindfulness has the positive impact on subjective well-being by means of the mediating effects of emotion regulation and resilience (Liu et al., 2015). For the social development, mindfulness predicts college students’ prosocial behaviors. For instance, college students with high trait mindfulness present less anger rumination and less online antisocial behavior (Liu et al., 2022) because of the impact of mindfulness on the cultivation of value-congruent behaviors (Monteiro et al., 2019), which tends to lessen harmful and undesirable tendencies toward others and potentially destructive impacts on society with a sense of kindness and compassion fostered. In China, private college students are generally active in social engagement and practice, but lazy in engaging in learning activities with weak self-discipline and without strong learning goals (Chen and Xiong, 2013), and even incapable of coordinating the relationship between participation in social activities and learning (Gu, 2007). It is indispensable for private college undergraduates to take active parts in mindfulness training to improve their self-awareness, self-management, and responsible decision-making that are import factors to the development of self-leadership. As Armstrong (2022) suggests, students are engaged in raising their awareness of the present moment by attentively focusing on some activity, such as mindfully breathing, openly monitoring the idea or feeling that may break concentration, and actively adopting an unbiased and curious attitude to what’s going on. As for teachers, they manage to introduce mindfulness into the class activities, and make full use of group discussion or brainstorming, for example, to discuss how college students manage their own time, how to respect others’ time, and realize the impact of time management on their own and others’ study and life, so as to improve their awareness of emotional management. Teachers also embed the mindfulness into the process of course teaching. When students work on learning activities which may stir up their challenging emotions, teachers can model students to take deep breaths before reaction, and offer students the space and time of pause and reflection. What’s more, teachers assist students to develop calm minds and calm bodies for the sake of increasing awareness and regulation of their own feelings and thoughts, which in turn can strengthen their compassion and empathy for peers and someone else, and thereby enable or create an inclusive and welcoming atmosphere and culture for the whole class and even for school.

This study shows that metacognitive skills are associated with student leadership development, which is similar to the previous studies (e.g., Black et al., 2016). In this study, metacognition also plays the mediating role in relationship between mindful agency and self-leadership. High-level metacognition is be conducive to exerting the influence of mindfulness on self-leadership. Private college undergraduates are also necessary to become effective metacognitive learners so that they could transfer the influence of mindfulness to the development of self-leadership. Metacognition is a cycle of planning, monitoring, regulation, and evaluation in process of students’ learning. First of all, private college students need to be aware of their weaknesses and strengths, on the basis of which they actively set goals and plan time for progress, and engage in learning with persistence and resilience or with help from teachers and peers. Besides, monitor learning and keep record of gain and loss based on which self-rewards or sanctions are given to regulating oneself. Then, self-evaluate this stage of learning and deploy the next phase of learning. Zimmerman (2002) also propose effective strategies of becoming a self-regulated and goal-oriented learner by setting specific proximal goals, making physical and social contexts compatible with goals, adopting suitable methods of achieving goals, efficiently managing and using time, self-evaluating the cause and effect of learning outcomes and adapting future strategies. Education workers are suggested encouraging private college students to pay attention to and accept their learning limitations by means of mindfulness training, and directing them obtain metacognitive skills to be self-regulated and goal-oriented, which would follow the progress in their self-leadership.

Although mindfulness cannot completely help to reduce negative emotions, it can contribute to changing the cognitive bias and increasing the positive cognitive reappraisal by regulating students’ metacognitive ability. Their self-reward and self-motivation can enhance their learning autonomy and competence. Education workers are advised to provide courses about mindfulness, metacognition and self-leadership or build self-leadership workshop for students, and make scientific design of self-leadership learning activities, such as on-site coaching, typical case analysis, self-leadership experience exchange, etc., so as to help college students form long-term and efficient self-leadership potential.

In this study, the direct effect of mindful agency is higher than the mediating effect to a large extent, and mindful agency may promote self-leadership through other ways. In addition to metacognitive ability, other mediating variables may also influence the relationship between mindful agency and self-leadership, such as the influence of emotional intelligence and self-efficacy on self-leadership (Lee, 2015). In the future research, it is necessary to explore other mediating variables to more comprehensively reveal the internal influencing mechanism of mindful agency on self-leadership.

## Data Availability Statement

The raw data supporting the conclusions of this article will be made available by the authors, without undue reservation.

## Ethics Statement

The studies involving human participants were reviewed and approved by the Yantai Nanshan University Ethics Committee. The patients/participants provided their written informed consent to participate in this study.

## Author Contributions

ZC: literature review, methodology, formal analysis, discussion, and writing. XZ: investigation, resources, and review and editing. Both authors have read and agreed to the published version of the manuscript.

## Conflict of Interest

The authors declare that the research was conducted in the absence of any commercial or financial relationships that could be construed as a potential conflict of interest.

## Publisher’s Note

All claims expressed in this article are solely those of the authors and do not necessarily represent those of their affiliated organizations, or those of the publisher, the editors and the reviewers. Any product that may be evaluated in this article, or claim that may be made by its manufacturer, is not guaranteed or endorsed by the publisher.
